# Childhood Leukemia, Military Aviation Facilities, and Population Mixing

**DOI:** 10.1289/ehp.112-a797

**Published:** 2004-10

**Authors:** Leo J. Kinlen

**Affiliations:** University of Oxford, Radcliffe Infirmary, Oxford, United Kingdom, E-mail: leo.kinlen@dphpc.ox.ac.uk

In a recent article on the striking cluster of childhood leukemia in 2000–2001 near the Fallon Naval Air Station in Nevada, Steinmaus et al. (2004) referred to the potential relevance of rural–urban population mixing. The population-mixing hypothesis was generated by the observation of excesses of childhood leukemia in two remote and isolated areas in Great Britain that had experienced influxes of significant numbers of workers as a result of the construction and operation of two large nuclear facilities (Kinlen 1988). Such mixing will increase the level of contacts between susceptible (more prevalent in rural areas) and infected individuals, promoting localized (frequently subclinical) epidemics of infections. If childhood leukemia is a rare response to a common—but unidentified—infection, then these localized epidemics will produce excess cases of the unusual complication, childhood leukemia.

Studies of all known examples of extreme rural–urban population mixing in Britain in the past 60 years have, in each instance, revealed significant temporary excesses of childhood leukemia (Kinlen 1995, 2000). These findings have been supported by studies conducted in other countries, most recently by an excess of childhood leukemia in isolated rural counties of the United States where substantial population increases have occurred (Wartenberg et al. 2004). None of these “mixing” situations, however, can compare in intensity with the indirect exposure of the small town of Fallon, Nevada (population 7,536), in only a few years, to over 100,000 military personnel from outside the area receiving training at the naval air station, reaching the extraordinary level of 55,000 in 2000 (GlobalSecurity.org 2003; . U.S. Navy 2002). That the world’s most sharply defined cluster of childhood leukemia (Alexander 1993; Steinmaus et al. 2004) should occur in association with the most extreme example of rural–urban population mixing could not be more arresting (Kinlen and Doll 2004).

Every opportunity should be taken to investigate the role that infection may have played in this extraordinary cluster of childhood leukemia. Unlike most studies of marked population mixing, where the relevant circumstances occurred some time ago, this recent cluster provides researchers with the chance to thoroughly study the cases (and other members of the population) for evidence of exposure to the relevant infectious agent. It is an opportunity that should not be missed.
